# 

**Published:** 1924-12

**Authors:** 


CHRISTMAS APPEALS

New Series. Vol. III. No. 39 (Vol. LXXIII. No. 1881). December, 1924. Monthly, Price 6d. (POST FREE 7½d.)

